# Novel Biomarkers of Mastitis in Goat Milk Revealed by MALDI-TOF-MS-Based Peptide Profiling

**DOI:** 10.3390/biology9080193

**Published:** 2020-07-28

**Authors:** Monica Matuozzo, Maria Stefania Spagnuolo, Hany A. Hussein, A. M. Gomaa, Andrea Scaloni, Chiara D’Ambrosio

**Affiliations:** 1 Institute for the Animal Production System in the Mediterranean Environment (ISPAAM), National Research Council (CNR), 80147 Naples, Italy; monica.matuozzo@gmail.com (M.M.); mariastefania.spagnuolo@cnr.it (M.S.S.); andrea.scaloni@ispaam.cnr.it (A.S.); 2 Department of Animal Reproduction and Artificial Insemination, National Research Centre, Giza 12622, Egypt; hnyhussein2@yahoo.com; 3 Department of Veterinary Research, Guangdong Haid Institute of Animal Husbandry and Veterinary (GHIAHV), Guangzhou 511400, China; 4 Animal Reproduction Research Institute (ARRI), Agriculture Research Center, Ministry of Agriculture, Giza 12556, Egypt; alaagomaa20@yahoo.com

**Keywords:** peptidomic, peptide profiling, mastitis, milk goat, biomarker

## Abstract

Mastitis is the most common infection of dairy goats impairing milk production and quality, which is usually recognized by mammary gland visual inspection and palpation. Subclinical forms of the disease are also widely represented, which lack the typical signs of the clinical ones but are still associated with reduced production and safety for human consumption of milk, generally presenting a high bacterial count. In order to obtain novel analytical tools for rapid and non-invasive diagnosis of mastitis in goats, we analyzed milk samples from healthy, subclinical and clinical mastitic animals with a MALDI-TOF-MS-based peptidomic platform, generating disease group-specific spectral profiles whose signal intensity and mass values were analyzed by statistics. Peculiar spectral signatures of mastitis with respect to the control were identified, while no significant spectral differences were observed between clinical and subclinical milk samples. Discriminant signals were assigned to specific peptides through nanoLC-ESI-Q-Orbitrap-MS/MS experiments. Some of these molecules were predicted to have an antimicrobial activity based on their strong similarity with homolog bioactive compounds from other mammals. Through the definition of a panel of peptide biomarkers, this study provides a very rapid and low-cost method to routinely detect mastitic milk samples even though no evident clinical signs in the mammary gland are observed.

## 1. Introduction

Goats were the earliest domesticated animal in the world, and large consumption of the corresponding meat and dairy products has characterized human habits overtime. Recently, the demand for goat milk and dairy products has increased in developing countries, because of their suitable physicochemical characteristics and beneficial effects on human health [1]. As a matter of the fact, goat milk was reported having reduced allergenicity and higher digestibility than the cow counterpart, and goat dairy products have been classified as functional foods based on their nutritional/dietetic properties [1,2,3]. In fact, goat milk was demonstrated to have an augmented representation of whey proteins and essential amino acids [4], increased levels of mono/poly-unsaturated fatty acids and medium-chain triglycerides [5], and a reduced content of lactose [2].

Taking into account the increased consumer demand, many efforts have recently been devoted to increase the amount of goat milk production worldwide, and to improve the quality of corresponding dairy products [6]. For example, animal dietary modifications have been introduced for increasing the functional properties of goat milk [7,8,9]. Moreover, particular care has been spent in preventing and managing the outcomes of animal mastitis, which represents the primary and most costly infection of dairy goats. In fact, mastitis determines a strong decrease in milk production and quality [10,11], reduces weight gain in lambs, and is the cause of culling for sanitary reasons [12]. Clinical mastitis is an inflammatory condition of the mammary gland that is caused by different microorganisms, mostly bacteria, but also by organ injury; generally, it is recognized during veterinary examination by visual inspection and palpation. Subclinical mastitis (SCM) forms also exist, which are due to coagulase-negative Staphylococci, and are about six-fold more common than the clinical ones [13]. They lack the typical above-mentioned mammary signs of the clinical form, but are still associated with a reduced production of milk, which also presents a high bacterial count and a reduced antioxidant content [14]. Accordingly, SCM forms are more difficult to be identified.

In cows, SCM has been associated with high somatic cell count (SCC) values in milk; the diagnostic value of this parameter is underlined by the importance EU directives gave in establishing precise legal limits of it [15]. Conversely, the SCC value in goat milk does not correlate with clinical and subclinical forms of mastitis [16,17,18]. As a matter of fact, goat milk naturally contains higher levels of somatic cells than cow milk; this is because milk secretion in goats is apocrine [19,20]. Indeed, cytoplasmic particles from the apical portion of secretory cells are physiologically shed in milk. As these particles are similar in size to milk somatic cells, they can be mistakenly counted as the latter [21,22]. Further, the SCC value is influenced by the animal lactation stage and lactation number. Indeed, SCC increases physiologically when the lactation stage progresses, and is higher in goats of higher parity. Thus, mastitis diagnosis in goat is made by evaluation of mammary clinical signs and/or bacteriological tests.

In the above-mentioned context, clinical observation, California mastitis test and white side test were the main field diagnostic tools used for mammary inflammation detection in bovine and goat, whereas culture and isolation were laboratory-based methods [23,24,25]. However, the outcome and interpretation of these diagnostic tests were neither reliable nor specific or confirmatory [26,27]. Recently, molecular diagnostics [28] including PCR [29], qRT-PCR [30], loop-mediated isothermal amplification [31,32], nucleotide sequencing [33] and lateral flow assays [34] were used for overcoming above-mentioned shortcomings and for specific diagnosis of mastitis in bovine and goat. However, accuracy, sensitivity and specificity remain the main concern for all such tests [25,35].

On the other hand, proteomics has been successfully used for the differentiation of healthy and mastitic bovine [36,37,38,39,40,41,42,43,44,45,46], ovine [47,48,49] and caprine [50,51] milk, describing the metabolic and defense response of the mammary gland to various pathogens/pathogen-related lipopolysaccharides. Depending on the case, proteomic analysis was performed either on milk fat globule and/or whey fraction, and allowed monitoring the pathophysiological status of the mammary gland, highlighting protein biomarkers to be used for the development of novel diagnostic assays. In some cases, protein expression differences between healthy individuals and those affected by clinical and subclinical mastitic forms were evidenced [43,46,47,48,51].

Differential analysis of the peptide content of biological fluids has been used to discover biomarkers for the diagnosis and monitoring of diseases; generally, these studies highlighted the higher diagnostic character of a panel of analytes, more than a single compound. Concomitant changes in the peptide profile were indicative of a trend toward or away from the disease state. In this context, different peptidomic studies on bovine milk were accomplished to discriminate healthy, subclinical and clinical mastitic individuals, proposing putative biomarker panels [40,45,52,53,54]. Based on their discovery character, these investigations were generally performed through a combination of chromatographic and MS procedures, often limiting the number of investigated samples.

Due to the need of novel analytical tools for a non-invasive and reliable diagnosis of mastitis in goat, and the lack of information on putative peptide biomarkers in this context, we analyzed milk samples from healthy, subclinical and clinical mastitic animals using a Matrix Assisted Laser Desorption Ionisation-Time of Fligh-Mass Spectrometry (MALDI-TOF-MS)-based peptidomic platform optimized to this purpose. We took advantage of our previous experience in a large screening of milk samples for speciation and adulteration detection purposes [55,56,57], generating disease group-specific milk spectral profiles. Statistical analysis of the latter ones allowed the identification of discriminant signals, based on their intensity and mass values. The latter were assigned to specific peptides through further nanoLC-ESI-Q-Orbitrap MS/MS experiments, which identified biomarker candidates of mastitis in goats.

## 2. Materials and Methods

### 2.1. Sample Collection and Preparation

A total of 72 milk samples were collected from 48 dairy goats of the Damascus (*n* = 24), and Anglo-Nubian (*n* = 24) breed located in Giza and Alexandria governorates, Egypt. All goats were in mid- to late-lactation at sampling. Animals were initially subjected to clinical and udder examination for the detection of abnormalities, which were suggestive for clinical mastitis [58]. Before sample collection, teats were disinfected with iodine pre-milking solution, dried with disposable paper towels, and wiped with cotton balls moist with 70% *v/v* ethanol. After the withdrawal of the first 3 to 4 squirts on the floor, a 10 mL milk sample was collected in a sterile tube from each udder half. Milk samples were kept at 4 °C and transferred immediately to the laboratory for the assessment of corresponding SCC values. Bacteriological examination was done within 24 h. Aliquots of samples were stored at −20 °C for further peptidomic analysis.

Animals were managed according to the local farm-production practices. All examinations were carried out kindly, and always by the same veterinarian, for avoiding animal suffering and stress.

### 2.2. Somatic Cell Count

SCC value in milk samples was determined with a NucleoCounter^®^ SCC-100™ instrument (ChemoMetec, Allerod, Denmark), which is based on ChemoMetec’s proven technology of Fluorescence image cytometry, using a single-use SCC-Cassette™ sampling and measuring device.

### 2.3. Bacteriological Examination

Milk samples were hand-mixed and opened in a biosafety level II cabinet. Bacteriological examination of milk samples was performed as recommended previously [59,60]. Briefly, 10 μL of milk were streaked by the quadrant streaking method over Blood Agar Base (bioMérieux, Warsaw, Poland), Mac Conkey Agar (BTL, Warsaw, Poland), Mannitol salt agar (Oxoid Ltd., Basingstoke, UK), and Edwards Medium (Oxoid Ltd., Basingstoke, UK) plates. Plates were incubated at 37 °C, and then read after 24 and 48 h. The bacteria were tentatively identified according to their cultural and morphological appearance, and Gram’s reaction [61]. Detailed identification of isolated bacteria was performed using standard biochemical tests and API tests (bioMérieux, Warsaw, Poland) [62,63].

### 2.4. Milk Amyloid A Titration

Milk amyloid A concentration was assessed by sandwich ELISA using a commercial kit (Tridelta Development Ltd., Wicklow, Leinster, Ireland), essentially according to the manufacturer’s instructions. Samples were diluted 1:50 *v/v* for the assay and analyzed in duplicate. The program GraphPad Prism 6 (GraphPad Software, San Diego, CA, USA) was used to perform two-way ANOVA, followed by the Tukey post-hoc test. *p* < 0.05 was considered significant.

### 2.5. MALDI-TOF-MS-Based Peptide Profiling

To obtain goat skimmed milk, initial milk samples were defatted by centrifugation at 4000× *g*, for 30 min, at 4 °C. Aliquots of the corresponding skimmed material (800 μL) were treated by adding 4 vol of cold acetone (−20 °C), and centrifuged at 4000× *g*, for 30 min, at 4 °C, to precipitate proteins and obtain solutions containing peptides [57]. Supernatants were vacuum dried and then solved in 0.1% TFA. For each sample, an aliquot (20 µL) was desalted and concentrated on a μC18 ZipTip (Millipore, Darmstadt, Germany) device, which was then eluted with 3 µL of 50% *v/v* acetonitrile, containing 0.1% *v/v* TFA. Samples were then added with 3 µL of matrix solution (25 mg/mL of α-ciano-4 hydroxycinnamic acid in 50% *v/v* acetonitrile, containing 0.1% TFA), spotted in quintuplicate (1 µL per spot) on an MSP 384 target ground steel plate (Bruker Daltonics, Bremen, Germany) and allowed to dry, at room temperature [56]. Spectral profiles were acquired by MALDI-TOF-MS using an UltraflexExtreme mass spectrometer (Bruker Daltonics, Bremen, Germany) equipped with the FlexControl software package (v 3.4, Bruker Daltonics, Bremen, Germany) [55]. Spectra were recorded in the positive linear mode (laser frequency, 1000 Hz; ion source 1 voltage, 25.2 kV; ion source 2 voltage, 22.5 kV; lens voltage, 8.50 kV; sample rate, 0.63; mass range, m/z 500–7000). Five independent spectra (1000 shots at random positions on the same target place, for spectrum) were automatically collected, externally calibrated by using the Peptide Calibration Standard 2 and Protein Calibration Standard 1 kit (Bruker Daltonics, Bremen, Germany), and subsequently analyzed. The above-mentioned instrument settings were maintained during the whole analysis of all milk samples with the aim of not compromising the recognition capability of the peptidomic platform.

FlexAnalysis (v 3.4) and ClinProt Tools (v 2.2) software packages (Bruker Daltonics, Bremen, Germany) were used for the analysis of all MALDI-TOF-MS data, which included spectral mass adjustment, optional smoothing (using the Savitsky-Golay algorithm with width 15 e cycles 2), spectral baseline subtraction, normalization, internal peak alignment, and peak picking. Pretreated data were then subjected to visualization and statistical analysis. Peaks showing a statistically significant difference in signal intensity or mass value were determined by means of Wilcoxon (PWKW), Anderson–Darling (PAD,) and t (PTTA) tests. A class prediction model was set up by Genetic Alghorithms (GA). Discriminant peaks were considered those presenting at least PAD *p*-value < 0.000001 a signal area/intensity fold change ratio ≥1.5 and ≤0.67. Finally, a principal component analysis (PCA) of the spectra was performed, which was carried out by an external MATLAB software tool integrated into ClinProt Tools software.

### 2.6. NanoLC-ESI-Q-Orbitrap MS/MS Analysis

Aliquots of each sample were subjected to desalting/concentration step on C18 ZipTip microcolumn (Millipore, Darmstadt, Germany) using 50% *v/v* acetonitrile, containing 5% *v/v* formic acid as eluent. Peptide mixtures were analyzed with an UltiMate 3000 HPLC RSLC nano system-Dionex coupled to a Q-ExactivePlus mass spectrometer through a Nanoflex ion source (Thermo Fisher Scientific, Waltham, MA, USA). Peptides were loaded on an Acclaim PepMapTM RSLC C18 column (150 mm × 75 μm ID, 2 μm particles, 100 Å pore size) (Thermo Fisher Scientific, Waltham, MA, USA), and eluted with a gradient of solvent B (19.9/80/0.1 *v/v/v* water/acetonitrile/formic acid) in solvent A (99.9/0.1 *v/v* water/formic acid), at a flow rate of 300 nL/min. The gradient of solvent B started at 3%, increased to 40% over 40 min, raised to 80% over 5 min, remained at 80% for 4 min, and finally returned to 3% in 1 min, with a column equilibrating step of 30 min before the subsequent chromatographic run. The mass spectrometer operated in data-dependent mode, using a full scan (m/z range 375–1500), nominal resolution of 70,000 automatic gain control target of 3,000,000, a maximum ion target of 50 ms, followed by MS/MS scans of the 10 most abundant ions. MS/MS spectra were acquired using a normalized collision energy of 32%, an automatic gain control target of 100,000, a maximum ion target of 100 ms, and a resolution of 17,500. A dynamic exclusion value of 30 s was also used. Two technical replicates were analyzed for each sample.

### 2.7. Database Search for Protein Identification

All MS and MS/MS raw data files per sample were merged for protein identification into Proteome Discoverer v 2.1 software (Thermo Scientific, Waltham, MA, USA), enabling the database search by Mascot algorithm v 2.4.2 (Matrix Science, London, UK). The following criteria were used: UniProtKB protein database (Capra hircus as taxonomy) including the most common protein contaminants, and oxidation of Methionine and pyroglutamate formation at N-terminal Glutamine as variable modifications. Peptide mass tolerance was set to ±10 ppm and fragment mass tolerance to ±0.05 Da. No proteolytic enzyme was set. Peptide candidates assigned based on a Mascot score ≥30 were considered confidently identified. Results were filtered to 1% false discovery rate.

### 2.8. Bioinformatics for Function Prediction

Identified peptides/proteins were in silico analyzed for their sequence with free available predictor software. The latter are based on SVM models that provide a prediction of molecular activity based on corresponding amino acid composition, sequence, peptide motifs, binary profile features, and physiochemical property information. Used web-based predictor software were the following: (i) Antinflam (http://metagenomics.iiserb.ac.in/antiinflam/pred.php) that recognizes anti-inflammatory peptides; (ii) CAMPR3 (http://www.camp3.bicnirrh.res.in) and Antimicrobial Peptide Scanner vr2 (https://www.dveltri.com/ascan/v2/ascan.html) that recognize antimicrobial peptides; (iii) dPABBs (http://ab-openlab.csir.res.in/abp/antibiofilm) that recognizes potential antibiofilm peptides; and (iv) AVPpred (http://crdd.osdd.net/servers/avppred/index.html) that recognizes potential antiviral peptides. The SVM model score threshold was set to 1 for prediction of anti-inflammatory molecules, and to 0.5 for antimicrobial and antibiofilm components; this value was set to 45 only in the case of antiviral peptides.

Peptide secondary structure prediction and helical wheel representation were obtained with PSIPRED 4.0 and MEMSAT-SVM software (http://bioinf.cs.ucl.ac.uk/psipred) and NetWheels software (http://lbqp.unb.br/NetWheels), respectively.

## 3. Results and Discussion

### 3.1. Milk Group Classification

**Figure 1:**
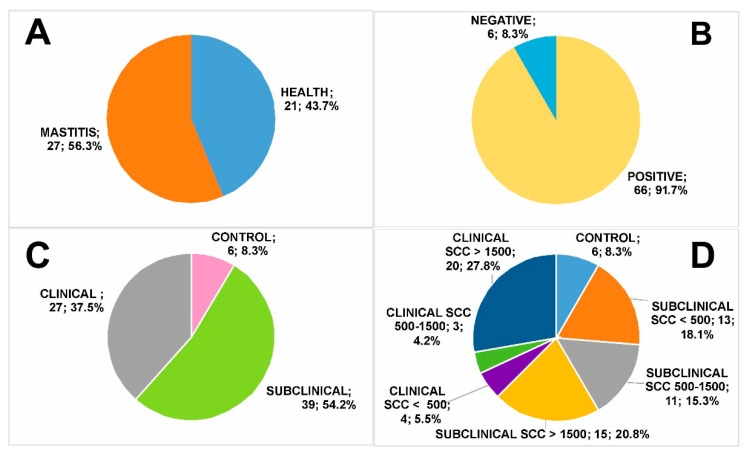
Classification of goat milk samples by different parameters. (**A**) Classification according to the clinical examination of goats; (**B**) classification according to bacteriological analysis of goat milk samples; (**C**) classification according to the combination of clinical examination and bacteriological analysis; (**D**) classification according to the combination of clinical examination, bacteriological analysis and evaluation of milk somatic cell count (SCC) values.

Clinical examination of the udder of 48 dairy goats revealed symptoms of clinical mastitis in 27 animals (43.7%), while 21 animals (56.3%) were clinically healthy and showed normal milk secretion (Figure 1A). Bacteriological examination performed on 72 milk samples revealed single/multiple positivities in 66 samples (91.7%), while 6 samples (8.3%) did not show any microbial growth (Figure 1B). Milk samples were then grouped according to results from the veterinary and bacteriological analysis. Therefore, 6 milk samples were assigned to the group of healthy animals showing negative bacteriological examination (8.3%), 39 samples were assigned to the group of healthy animals showing at least one positive bacteriological examination (54.2%), and 27 samples were assigned to the group of animals with clinical mastitis (37.5%) (Figure 1C).

Although somatic cells are normally present in goat mammary secretions, their value increases significantly as a consequence of intramammary infection. Therefore, SCC evaluation was also carried out as a further index of the udder health status [64] and of the hygienic quality of milk [65]. It is worth mentioning that the legislation of international dairy food has fixed a specific value of SCC in bovine milk to distinguish healthy samples from unhealthy ones [66]. We already mentioned that the SCC value in goat milk is influenced by several factors, such as breed, stage of lactation [23], type of birth, estrus [67], diurnal, monthly, and seasonal variations [68]. Indeed, the relationship between SCC and mastitis infection has not been established and, accordingly, defined by a dedicated law. As an international and unambiguous legislative limit for goat milk is not available yet and various SCC values have been reported in the literature [69,70,71], we chose to classify sample groups also based on corresponding SCC values. Accordingly, we classified as healthy (control) samples as those from animals not showing clinical signs and negative bacteriological tests; they all had an SCC value < 500 × 10^3^ cells/mL [72,73,74,75] (Figure 1D). Milk samples from animals not showing clinical signs but having at least a positive value of bacteriological examination, and variable SCC values were classified as subclinical, because co-association of a positive bacteriological investigation was correlated to the presence of an ongoing infection that was not manifested yet (Figure 1D). Finally, milk samples from animals having clinical signs and positive values of bacteriological examination, and variable SCC values were classified as clinical (Figure 1D). Subclinical and clinical groups were then divided into subgroups based on SCC values < 500 × 10^3^ cells/mL, within the range 500–1500 × 10^3^ cells/mL, and >1500 × 10^3^ cells/mL, to yield final sample grouping reported below and in Figure 1D.

- Healthy—no clinical signs, negative bacteriological tests and SCC < 500 × 10^3^ cells/mL (control, *n* = 6, 8.3% of total);
- Subclinical mastitis—no clinical signs, positive bacteriological tests (low SCC, SCC < 500 × 10^3^ cells/mL, *n* = 13, 18.1% of total; medium SCC, SCC = 500–1500 × 10^3^ cells/mL, *n* = 11, 15.3% of total; high SCC, SCC > 1500 × 10^3^ cells/mL, *n* = 15, 20.8% of total);
- Clinical mastitis—evident clinical signs and positive bacteriological tests (low SCC, SCC < 500 × 10^3^ cells/mL, *n* = 4, 5.5% of total; medium SCC, SCC = 500–1500 × 10^3^ cells/mL, *n* = 3, 4.2% of total; high SCC, SCC > 1500 × 10^3^ cells/mL, *n* = 20, 27.8% of total).

The seven groups were then prepared for further peptidomic analysis based on MALDI-TOF-MS experiments.

### 3.2. MALDI-TOF-MS Peptide Profiling

**Figure 2:**
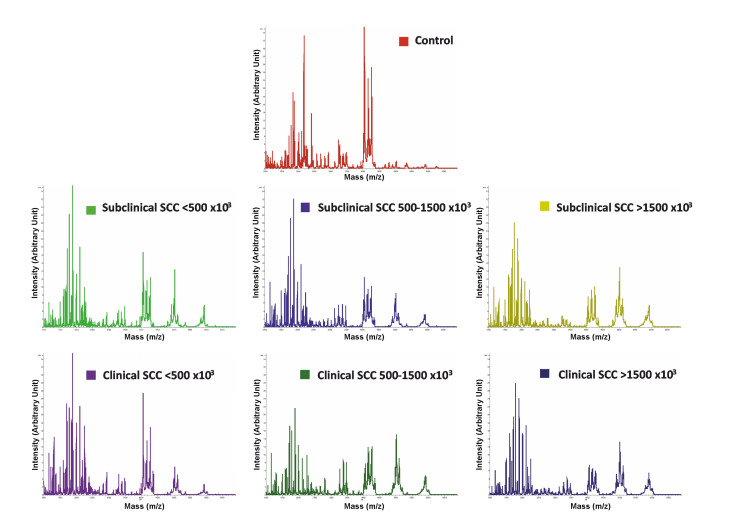
Average MALDI-TOF mass spectra of control milk samples (red), subclinical mastitic milk samples with SCC values < 500 × 10^3^ cells/mL (green), subclinical mastitic milk samples with SCC values = 500–1500 × 10^3^ cells/mL (blue), subclinical mastitic milk samples with SCC values > 1500 × 10^3^ cells/mL (apple green), clinical mastitic milk samples with SCC values < 500 × 10^3^ cells/mL (violet), clinical mastitic milk samples with SCC values = 500–1500 × 10^3^ cells/mL (dark green), clinical mastitic milk samples with SCC values > 1500 × 10^3^ cells/mL (dark blue).

In order to identify peptide markers associated with above-mentioned subclinical and clinical classification, also considering SCC sub-classification, milk samples from healthy and affected animals were skimmed, removed for proteins and analyzed in linear mode by MALDI-TOF-MS [55,56,57]; this allowed a rapid detection of the corresponding spectral profile signatures. To ensure optimal MALDI-TOF-MS reproducibility and sample discrimination accuracy, skimmed milk samples were loaded in quintupled on a steel target instrument plate and analyzed in technical quintuplicate. As an example, mass spectra obtained for a control (A) and a clinical with SCC > 1500 × 10^3^ cells/mL (B) milk sample are reported in Figure S1. The acquired mass spectra were normalized by importing raw data to dedicated software (ClinProt, Bruker Daltonics, Bremen, Germany), and any signal-to-noise ratio intensity beyond 5:1 was considered as a peak. Representative average mass spectra for each sample group are shown in Figure 2.

Statistical analysis of all MALDI-TOF mass spectra was then performed; based on signal intensity, 47 peaks were identified as showing significant differences among different sample groups (PAD, *p* < 0.000001). The above-mentioned peaks were then analyzed for signal intensity changes; the ones displaying a significant higher (fold change ≥1.5) or lower intensity (fold change ≤0.67) with respect to the control group were finally selected. A total of 45 peaks (ranging from m/z 1153.17 to 6279.61) emerged in subclinical and clinical samples as showing significant intensity changes (Table 1). In particular, 14 average signals (m/z 1153.17, 1307.03, 1703.72, 1720.73, 1837.78, 2181.62, 2195.89, 4162.48, 4264.35, 5017.09, 5107.34, 5192.21, 5914.71 and 6001.46) showed common increasing (9 in number) or decreasing (5 in number) intensity trends in all clinical and subclinical forms, with respect to the control. All of them did not depend on the ascertained SCC value; thus, may represent good molecular biomarker candidates for future dedicated studies.

**Figure 3:**
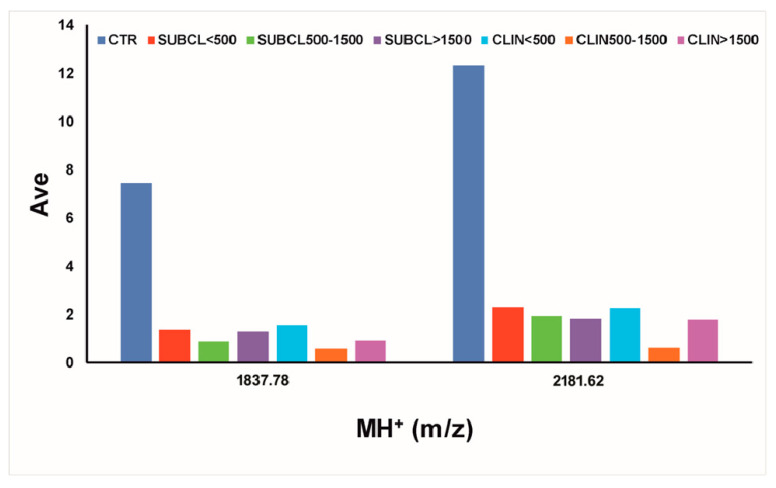
Average intensity trends of serum amyloid A3 peptide markers identified by MALDI-TOF-MS profiling of subclinical, clinical and control milk samples.

For example, Figure 3 illustrates two of the significant average signals displaying a decreasing trend in all subclinical and clinical groups, compared to control.

On the other hand, 18 average signals (m/z 1491.76, 1602.67, 1621.69, 1784.82, 1853.38, 2000.24, 2295.14, 2928.88, 3270.31, 3293.95, 3407.20, 3849.31, 4054.94, 4810.20, 4922.04, 5353.01, 5828.20 and 6279.61) showed common and coherent increasing (7 in number) or decreasing (11 in number) intensity trends in both clinical and subclinical forms having the same SCC cataloging, with respect to control (Table 1); among those, 8 showed a common and coherent increasing (2 in number) or decreasing (6 in number) intensity changes in samples having at the same SCC = 500–1500 × 10^3^ cells/mL and >1500 × 10^3^ cells/mL. More importantly, no average signals showing common quantitative trends among all SCC subgroups allowed discrimination between clinical and subclinical forms (Table 1). As expected, PCA of all the data was in line with the recognition capability values highlighted above and in Table 1 (data not shown); a good separation of the data was evident only in the case of healthy samples. Due to a higher number of signals in the mass spectra, our results were suggestive of increased activity of proteases in both subclinical and clinical milk samples, with respect to the healthy ones. These findings were in good agreement with previous dedicated studies on various milk samples from different mammals [76,77], which also detected an increased representation in mastitic material of hydrolytic enzymes from bacteria and cells involved in inflammatory processes [78,79].

Average signals related to MALDI-TOF-MS intensity changes between various groups were further investigated for corresponding molecular species. In particular, nanoLC-ESI-Q-Orbitrap MS/MS analysis of milk samples and database search of resulting data were used for peptide assignment; results are reported in Table 2. Based on their number, MALDI-TOF-MS varying average signals were, in order, associated with fragments from β-casein, serum amyloid A3, αs1- and αs2-casein, respectively. In particular, MALDI-TOF-MS average signal intensity changes suggested a significant production in subclinical and clinical mastitic goat milk of peptide fragments resulting from proteolysis of β-casein, as already observed in the bovine counterpart disease models [40,45,52,53]. In general, their nature well paralleled the one reported in above-mentioned studies, with small and large peptides originating from protein C-terminus, i.e., (197–207), (195–206), (193–206), (192–206), (192–207), (190–205), (191–207), (188–207) and (163–206), (162–206), (160–205)/(161–206), (161–207), (154–206), (154–207), showing an augmented representation. In particular, identical or very similar homologs of peptides (197–207), (193–206), (192–206), (192–207), (190–205), (191–207) and (188–207) were already identified by more accurate quantitative methods as a biomarker of disease in subclinical [53] and clinical [40,45,52] bovine mastitis. Some of these peptides were previously characterized for their antimicrobial properties against Gram-negative bacteria [80] or immunomodulatory action toward macrophages from germ-free or from human flora-associated mice [81]. As it concerns peptides (163–206), (162–206), (160–205)/(161–206), (161–207), (154–206) and (154–207), C-terminal truncated bovine homologs have already been identified with increased quantitative levels in clinical mastitis [40,45]. Finally, the decreased levels of peptides (182–207), (177–205), (177–206), (178–207), (177–207), (171–206), (170–206) and (170–207) measured in subclinical and clinical mastitic goat milk were suggestive of an increased protease activity favoring their degradation toward above-mentioned shorter molecular form.

On the other hand, MALDI-TOF-MS-based peptide profiling experiments indicated that all fragments from serum amyloid A3, i.e., (19–35) and (19–37), were down-represented in subclinical and clinical mastitis goat milk samples, notwithstanding their SCC value, thus suggesting reduced proteolysis of this protein after disease outcome. Variably represented fragments originating from proteolysis of serum amyloid A have already been reported in bovine milk from an experimental model of *Streptococcus uberis* mastitis [45], but none of the previously ascertained molecules matched the ones described here for infected goat milk. This may be due to the different experimental approach authors used for quantitative peptidomic analysis (MALDI-TOF-MS vs nanoLC-ESI-MS/MS) but also to subtle sequence differences present between bovine and goat serum amyloid A3. This protein is one of the major acute-phase effectors in ruminants [82,83].

Conversely, our profile measurements on as1-casein-derived peptides (21–32), (16–47) and (16–48) in clinical mastitic goat milk found a good parallel with quantitative data through more accurate methods on bovine milk counterparts [40,45,53], which proved the concomitant over-representation the smaller molecular homologs and down-representation of the larger parental compound species. In this case, some bovine peptide counterparts were proved to have antimicrobial activity against Gram-positive and Gram-negative bacteria, and yeasts [84]. Finally, the decreased representation of αs2-casein-derived peptides (199–208) and (190–208) in mastitic goat samples was in good agreement with quantitative results from bovine disease models [45], and was suggestive on an increased degradation of this protein in diseased animals.

### 3.3. Determination of Milk Amyloid A

The acute phase reaction is an element of nonspecific resistance; it is associated with the increase of specific proteins that are recognized as a marker of inflammation in mammals. Milk amyloid A (MAA) is considered a reliable and sensitive marker of mastitis [85] because its concentration significantly increases following mammary glands infection in ewe [49,86] and cows [38,39,43,87,88], as a result of protein leakage from the blood to the milk and as mammary glands epithelial cell-response to infection [89,90]. Therefore, MAA concentration was measured in goat milk samples from subclinical and clinical groups with the aim to evaluate the occurrence of this phenomenon also in goats, and to ascertain whether corresponding protein levels correlated with the abundance of the identified peptides. Protein levels of control samples were similar to those of subclinical and clinical ones having similar SCC values.

**Figure 4:**
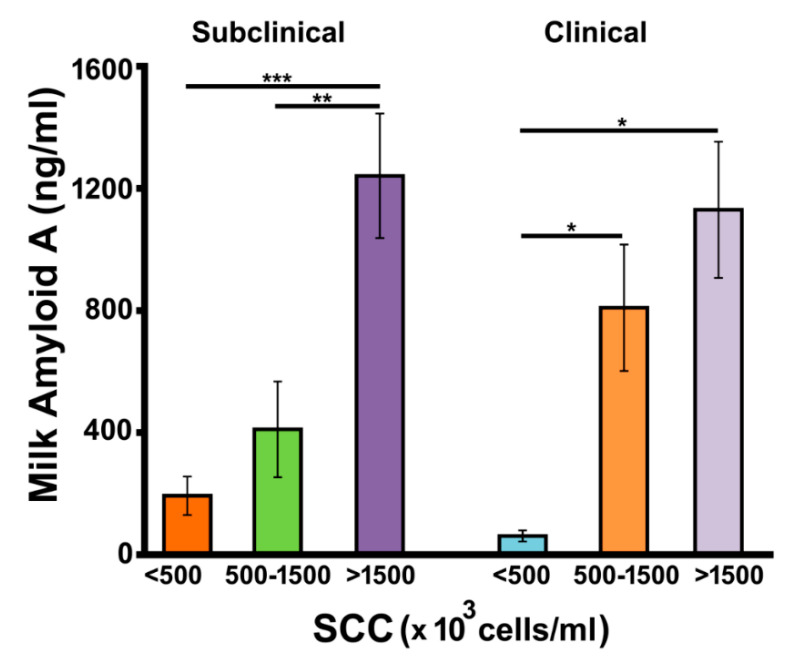
Determination of milk amyloid A in mastitic goat milk samples. Protein from subclinical (left) and clinical (right) samples with different SCC was titrated by sandwich ELISA according to what reported in the experimental section. Samples were analyzed in duplicate and data are reported as mean values ± SEM. The program GraphPad Prism 6 (GraphPad Software, San Diego, CA, USA) was used to perform two-way ANOVA, followed by the Tukey post-hoc test. * *p* < 0.05; ** *p* < 0.01; *** *p* < 0.001.

As expected, subclinical mastitis samples with SCC > 1500 × 10^3^ cells/mL showed MAA concentration values significantly higher than that of both subclinical with SCC = 500–1500 × 10^3^ cells/mL (*p* < 0.01) and subclinical with SCC < 500 × 10^3^ cells/mL counterparts (*p* < 0.001) (Figure 4), with corresponding protein titer paralleling SCC value. Similarly, clinical mastitis samples with SCC > 1500 × 10^3^ cells/mL or SCC = 500–1500 × 10^3^ cells/mL showed MAA levels higher than samples with SCC < 500 × 10^3^ cells/mL (*p* < 0.05). Again, protein concentration values paralleled SCC ones. Moreover, no great differences between subclinical and clinical samples with the same SCC count were observed (Figure 4).

Interestingly, the amount of MAA measured in mastitic milk samples having different SCC values was found to be negatively associated, although not significantly, with corresponding levels of MAA peptides ascertained by MALDI-TOF-MS analysis, thus suggesting the hampering of degradation phenomena affecting this protein in order to maintain its augmented molecular levels during infection.

### 3.4. Prediction of Proteases Generating Milk Peptides Ascertained in Mastitis

**Figure 5:**
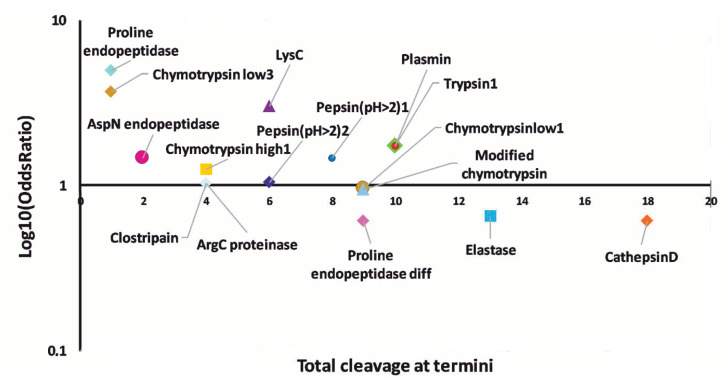
Evaluation of the various proteolytic enzymes putatively involved in the release of the milk peptides. A logarithmic scatter plot graph plotted using odds ratio (*X*-axis) and the total sites cleaved by the enzyme at termini (*Y*-axis).

Deregulated peptides here identified by combined peptidomic experiments were further subjected to bioinformatic analysis to identify proteases involved in the corresponding molecular release. This analysis was based on the evaluation of amino acids occurring at peptide N-terminal/C-terminal regions as well as on known specificity of proteolytic enzymes (Table S1). As shown in Figure 5, cathepsin D, elastase, trypsin-like, plasmin and chymotrypsin were predicted as the ones highly involved in the release of peptide fragments. Indeed, various proteolytic enzymes with such substrate specificity were already identified in mastitic bovine and sheep milk [91,92], and were predicted to be involved in the generation of bovine homologs of the peptides reported in this study [40,53].

### 3.5. Peptide Function Prediction

Identified peptides were then analyzed by bioinformatics in order to predict their putative activity and, consequently, whether they should play a physiological function. In the literature, it is well known that peptides deriving from the hydrolysis of caseins may present multiple activities [93,94].

As reported in Table 3, several peptides showed potential antimicrobial, antiviral and anti-inflammatory properties. Our results agree with those obtained in some peptidomic studies conducted on bovine mastitis [52], in which similar functions were predicted for homologous peptides. In order to corroborate this prediction, all deregulated peptides identified in this study were further subjected to bioinformatic analysis for recognizing their possible tendency to generate an amphipathic helix in a membrane-like environment. Figure S2 shows those for which an antimicrobial activity was predicted, all of which also showed an amphipathic character. 

## 4. Conclusions

Mastitis is associated with a significant impairment of milk quality and production. To date, pathology diagnosis in goats occurs essentially by evaluation of clinical signs and/or bacteriological examination. Therefore, development of novel analytical tools for a rapid and non-invasive diagnosis of mastitis in goats are strongly encouraged. In this study, MALDI-TOF-MS-based peptidomic profiling method of goat milk allowed discriminating between healthy and subclinical/clinical mastitic samples, defining a panel of peptide biomarkers useful to this purpose. This molecular panel may also eventually be used for early diagnosis of subclinical mastitis in goats, before the onset of the pathology at the clinical level, and regardless of the value of the somatic cells present in milk samples. Conversely, this approach did not differentiate clinical and subclinical samples. Above-mentioned peptides were molecular homologs of compounds already identified as candidate disease biomarkers for bovine mastitis [40,45,52,53]; some of them were previously proved to elicit a significant antimicrobial activity. They generally derived from an increased proteolytic activity in mastitic goat milk, in agreement to what was already detected in this and other mammals.

Whenever dedicated instruments and well-experienced personnel are available, the use of the rapid and low-cost analytical procedure reported in this study may help in recognizing the occurrence of mastitis in goat milk samples, even though no evident clinical signs in the mammary gland of corresponding animals are observed. In fact, the whole analytical workflow (from initial sample processing to MALDI-TOF-MS data analysis output) it is no more than an hour-long, and the cost of the reagents (excluding instrument service) is nowadays negligible. At present, the technology proposed here cannot substitute classical bacteriological tests for mastitis detection, although it can be easily integrated with some of them. In the future, being based on the definition of a peptide biomarker panel, we believe it will open the way to the development of novel, highly informative immunoassays focused on the combined, simultaneous evaluation of multiple species.

## Figures and Tables

**Table 1:** MALDI-TOF-MS profiling data of peptides from subclinical (S) and clinical (CL) goat milk groups with respect to corresponding control (C) one. Mass values refer to m/z (Da); DAve values correspond to the difference between the maximal and the minimal average peak area/intensity of all classes, respectively. PTTA, PWKW and PAD values correspond to the *p*-values of *t*-test, Wilcoxon test, and Anderson–Darling test, respectively. Ave values correspond to the peak area/intensity average values of all classes. Fold change values correspond to the Ave values ratio between each class and control (C) class. Fold change values ≥ 1.5 and ≤ 0.67 are reported as highlighted in red and blue color; those showing common and coherent increasing or decreasing trends in both clinical and subclinical forms having the same SCC cataloging are highlighted in corresponding darker colors. S < 500, subclinical samples with SCC values < 500×10^3^ cells/mL; S = 500 −1500, subclinical samples with SCC values within the range 500–1500 × 10^3^ cells/mL; S > 1500, subclinical samples with SCC values > 1500 × 10^3^ cells/mL; CL < 500, clinical samples with SCC values < 500 × 10^3^ cells/mL; CL = 500–1500, clinical samples with SCC values within the range 500–1500 × 10^3^ cells/mL; CL > 1500, clinical samples with SCC values > 1500 × 10^3^ cells/mL.

Mass Value	DAve	PTTA	PWKW	PAD	Ave							Fold Change					
					CTR	S < 500	S = 500–1500	S > 1500	CL < 500	CL = 500–1500	CL > 1500	S < 500/C	S = 500–1500/C	S > 1500/C	CL < 500/C	CL = 500–1500/C	CL > 1500/C
1053.44	0.63	0.00252	0.000215	<0.000001	1.52	1.23	1.59	1.44	0.97	1.24	1.11	0.81	1.05	0.95	0.64	0.82	0.73
1153.17	2.98	<0.000001	<0.000001	<0.000001	1.62	2.64	4.6	3.85	3.89	4.11	2.53	1.63	2.84	2.38	2.40	2.54	1.56
1210.03	1.66	0.000035	0.000421	<0.000001	2.32	1.57	2.94	2.67	3.18	1.51	1.55	0.68	1.27	1.15	1.37	0.65	0.67
1266.79	1.48	0.0000054	0.0000122	<0.000001	1.15	2.03	2.04	1.58	2.63	1.54	1.25	1.77	1.77	1.37	2.29	1.34	1.09
1307.03	4.93	<0.000001	<0.000001	<0.000001	0.83	1.52	2.87	2.61	5.76	2.41	1.51	1.83	3.46	3.14	6.94	2.90	1.82
1491.76	2.51	0.00000958	0.00478	<0.000001	1.96	3	4.47	3.24	3.34	2.43	4.01	1.53	2.28	1.65	1.70	1.24	2.05
1602.67	3.83	<0.000001	<0.000001	<0.000001	2.02	3.61	4.03	5.16	3.63	2.81	5.85	1.79	2.00	2.55	1.80	1.39	2.90
1621.69	2.47	<0.000001	<0.000001	<0.000001	2.29	3.87	3.94	4.63	2.16	2.6	4.55	1.69	1.72	2.02	0.94	1.14	1.99
1703.72	6.07	<0.000001	<0.000001	<0.000001	2.13	3.66	5.5	5.57	8.2	4.83	6.26	1.72	2.58	2.62	3.85	2.27	2.94
1720.73	3.99	0.0000617	0.0063	<0.000001	4.01	8.01	7.25	7.64	6.8	6.3	7.52	2.00	1.81	1.91	1.70	1.57	1.88
1784.82	5.93	<0.000001	0.0000715	<0.000001	5.77	11.54	11.7	10.37	7.96	6.01	10.8	2.00	2.03	1.80	1.38	1.04	1.87
1837.78	6.87	<0.000001	<0.000001	<0.000001	7.47	1.37	0.92	1.29	1.57	0.6	0.96	0.18	0.12	0.17	0.21	0.08	0.13
1853.38	5.2	<0.000001	<0.000001	<0.000001	5.43	3.52	2.52	2.64	6.7	1.5	1.5	0.65	0.46	0.49	1.23	0.28	0.28
1884.73	9.36	<0.000001	0.00129	<0.000001	8.43	17.1	14.68	9.87	14.13	7.75	10.09	2.03	1.74	1.17	1.68	0.92	1.20
2000.24	3.07	0.0589	0.000779	<0.000001	3.36	5.74	4.95	5.34	6.44	4.52	5.92	1.71	1.47	1.59	1.92	1.35	1.76
2110.71	5.18	<0.000001	0.0168	<0.000001	5.78	8.71	6.84	4.53	8.31	3.53	6.8	1.51	1.18	0.78	1.44	0.61	1.18
2181.62	11.74	<0.000001	<0.000001	<0.000001	12.36	2.32	1.94	1.86	2.26	0.62	1.79	0.19	0.16	0.15	0.18	0.05	0.14
2195.89	7.58	<0.000001	<0.000001	<0.000001	8.43	3.1	2.62	2.68	3.49	0.84	2.17	0.37	0.31	0.32	0.41	0.10	0.26
2257.88	2.7	0.123	0.201	<0.000001	4.46	4.68	4.11	4.01	6.71	4.02	4.06	1.05	0.92	0.90	1.50	0.90	0.91
2295.14	2.64	<0.000001	<0.000001	<0.000001	4.04	3.15	2.6	1.41	2.95	2.06	1.64	0.78	0.64	0.35	0.73	0.51	0.41
2670.72	1.06	<0.000001	0.0000017	<0.000001	0.98	1.17	1.48	1.44	1.15	2.04	1.64	1.19	1.51	1.47	1.17	2.08	1.67
2812.25	1.38	0.000173	0.00122	<0.000001	1.69	1.54	1.49	1.85	1.59	2.87	1.64	0.91	0.88	1.09	0.94	1.70	0.97
2928.88	1.76	<0.000001	<0.000001	<0.000001	2.52	2.03	1.3	0.76	2.3	0.75	0.91	0.81	0.52	0.30	0.91	0.30	0.36
3270.31	2.57	0.00393	0.000858	<0.000001	4.48	2.17	3.01	2.41	1.91	3.41	2.62	0.48	0.67	0.54	0.43	0.76	0.58
3293.95	1.15	0.00048	0.0000715	<0.000001	2.41	1.93	1.56	1.48	2.53	1.38	1.38	0.80	0.65	0.61	1.05	0.57	0.57
3382.47	2.47	0.00000102	0.0000715	<0.000001	3.14	2.27	3.34	1.79	2.02	4.26	3.07	0.72	1.06	0.57	0.64	1.36	0.98
3407.2	0.96	0.0000111	0.00000944	<0.000001	2.27	1.4	1.58	1.31	1.77	1.76	1.3	0.62	0.70	0.58	0.78	0.78	0.57
3481.56	2.92	<0.000001	<0.000001	<0.000001	3.37	3	3.2	1.06	2.63	3.97	2.26	0.89	0.95	0.31	0.78	1.18	0.67
3693.69	0.49	<0.000001	<0.000001	<0.000001	0.74	0.73	0.46	0.37	0.49	0.86	0.57	0.99	0.62	0.50	0.66	1.16	0.77
3849.31	0.5	<0.000001	<0.000001	<0.000001	0.69	0.85	0.42	0.35	0.62	0.79	0.41	1.23	0.61	0.51	0.90	1.14	0.59
3944.68	0.88	0.0267	0.0798	<0.000001	0.82	1.46	0.64	0.62	1.04	0.58	0.77	1.78	0.78	0.76	1.27	0.71	0.94
4054.94	12.51	<0.000001	<0.000001	<0.000001	17.37	9.05	7.22	4.97	13.01	4.86	5.99	0.52	0.42	0.29	0.75	0.28	0.34
4162.48	6.99	0.00000828	0.0000379	<0.000001	12.74	7.07	7.07	6.68	7.86	7.49	5.76	0.55	0.55	0.52	0.62	0.59	0.45
4264.35	9.27	<0.000001	<0.000001	<0.000001	14.59	7.77	8.17	6.48	9.33	8.09	5.32	0.53	0.56	0.44	0.64	0.55	0.36
4356.77	2.89	0.000803	0.000175	<0.000001	1.38	1.35	2.53	1.66	4.24	2.86	1.73	0.98	1.83	1.20	3.07	2.07	1.25
4810.2	0.94	<0.000001	0.0000168	<0.000001	0.88	0.95	0.49	1.32	0.38	0.55	0.97	1.08	0.56	1.50	0.43	0.63	1.10
4922.04	4.58	<0.000001	<0.000001	<0.000001	0.81	3.73	2.32	5.39	0.9	2.34	4.35	4.60	2.86	6.65	1.11	2.89	5.37
5017.09	9.67	<0.000001	<0.000001	<0.000001	1.53	8.77	7.38	11.09	3.84	9.88	11.2	5.73	4.82	7.25	2.51	6.46	7.32
5107.34	5.77	<0.000001	<0.000001	<0.000001	0.35	2.63	3.56	5.88	3.34	6.08	6.12	7.51	10.17	16.80	9.54	17.37	17.49
5192.21	1.5	<0.000001	<0.000001	<0.000001	0.15	0.63	0.91	1.65	0.88	1.47	1.59	4.20	6.07	11.00	5.87	9.80	10.60
5353.01	0.55	<0.000001	<0.000001	<0.000001	0.86	0.83	0.66	0.31	0.59	0.51	0.31	0.97	0.77	0.36	0.69	0.59	0.36
5828.2	2.11	<0.000001	<0.000001	<0.000001	0.42	1.8	1.34	2.53	0.52	1.48	2.24	4.29	3.19	6.02	1.24	3.52	5.33
5914.71	4.01	<0.000001	<0.000001	<0.000001	0.76	3.98	3.31	4.77	1.6	3.95	4.74	5.24	4.36	6.28	2.11	5.20	6.24
6001.46	1.55	<0.000001	<0.000001	<0.000001	0.17	0.72	0.94	1.66	0.68	1.15	1.72	4.24	5.53	9.76	4.00	6.76	10.12
6279.61	0.35	<0.000001	<0.000001	<0.000001	0.48	0.36	0.25	0.13	0.18	0.2	0.15	0.75	0.52	0.27	0.38	0.42	0.31

**Table 2:** Deregulated peptides identified by nanoLC-ESI-Q-Orbitrap MS/MS procedures. Experimental MALDI-TOF-MS (average—Av) mass values, theoretical (average—Av and monoisotopic—Mi) mass values, experimental (monoisotopic) nanoLC-ESI-Q-Orbitrap m/z and charge values, amino acid sequence, parental protein names, protein accession, protein fragment assignment and modifications are reported. Mox, oxidized methionine; pGlu, N-terminal pyroglutamic acid.

Exp. MALDI MH^+^ Value (Av)	Theor. MH^+^ Value (Av)	Theor. MH^+^ Value (Mi)	Exp. Nanolc-ESI-Q-Orbitrap m/z	Charge	Peptide Sequence	Parental Protein	Accession	Fragment
1053.44	1053.28	1052.62	526.81	2	LGPVRGPFPI	β-casein	P33048	196–205
1153.17	1152.42	1151.69	576.35	2	GPVRGPFPILV	β-casein	P33048	197–207
1210.03	1210.41	1209.66	605.33	2	TNAIPYVRYL	αs2-casein	P33049	199–208
1266.79	1265.58	1264.77	632.89	2	VLGPVRGPFPIL	β-casein	P33048	195–206
1307.03	1305.43	1304.65	652.83	2	INHQGLSPEVPN	αs1-casein	NP_001272624.1	21–32
1491.76	1491.81	1490.87	745.94	2	EPVLGPVRGPFPIL	β-casein	P33048	193–206
1602.67	1602.91	1601.9	801.45	2	QEPVLGPVRGPFPIL	β-casein	P33048	192–206 pGlu
1621.69	1619.94	1618.92	809.46	2	QEPVLGPVRGPFPIL	β-casein	P33048	192–206
1703.72	1702.04	1700.97	850.98	2	QEPVLGPVRGPFPILV	β-casein	P33048	192–207 pGlu
1720.73	1719.07	1717.99	859.5	2	QEPVLGPVRGPFPILV	β-casein	P33048	192–207
1784.82	1783.12	1781.99	891.50	2	LYQEPVLGPVRGPFPI	β-casein	P33048	190–205
1837.78	1836.03	1834.85	917.93	2	QGWGTFLREAGQGAKDM	serum amyloid A3	ABQ51197.1	19–35 pGlu
1853.38	1852.03	1850.84	925.92	2	QGWGTFLREAGQGAKDM	serum amyloid A3	ABQ51197.1	19–35 M^ox^, pGlu
1884.73	1882.25	1881.06	941.04	2	YQEPVLGPVRGPFPILV	β-casein	P33048	191–207
2000.24	2001.42	2000.19	500.80	4	SLSQPKVLPVPQKVVPQR	β-casein	P33048	164–181(A^177^→V)
2110.71	2108.57	2107.22	1054.12	2	LLYQEPVLGPVRGPFPILV	β-casein	P33048	189–207
2181.62	2178.43	2177.03	726.34	3	QGWGTFLREAGQGAKDMWR	serum amyloid A3	ABQ51197.1	19–37 pGlu
2195.89	2194.43	2193.02	731.68	3	QGWGTFLREAGQGAKDMWR	serum amyloid A3	ABQ51197.1	19–37 M^ox^, pGlu
2257.88	2255.74	2254.29	1127.66	2	FLLYQEPVLGPVRGPFPILV	β-casein	P33048	188–207
2295.14	2294.72	2293.21	765.07	3	AMKPWTQPKTNAIPYVRYL	αs2-casein	P33049	190–208 M^ox^
2670.72	2665.23	2663.53	888.52	3	PIQAFLLYQEPVLGPVRGPFPILV	β-casein	P33048	184–207
2812.25	2812.43	2810.56	1405.78	2	MPIQAFLLYQEPVLGPVRGPFPILV	β-casein	P33048	183–207 M^ox^
2928.88	2927.52	2925.59	975.87	3	DMPIQAFLLYQEPVLGPVRGPFPIIV	β-casein	P33048	182–207 Mox
3270.31	3266.87	3264.75	1088.92	3	AVPQRDMPIQAFLLYQEPVLGPVRGPFPI	β-casein	P33048	177–205 M^ox^
3293.95	3294.92	3292.78	1098.27	3	VVPQRDMPIQAFLLYQEPVLGPVRGPFPI	β-casein	P33048	177–205(A^177^→V) M^ox^
3382.47	3380.03	3377.84	1126.62	3	AVPQRDMPIQAFLLYQEPVLGPVRGPFPIL	β-casein	P33048	177–206 M^ox^
3407.2	3407.06	3404.85	1135.96	3	VPQRDMPIQAFLLYQEPVLGPVRGPFPILN	β-casein	P33048	178–207(V^207^→N)
3481.56	3479.16	3476.91	1159.64	3	AVPQRDMPIQAFLLYQEPVLGPVRGPFPIL	β-casein	P33048	177–207 M^ox^
3693.69	3696.28	3693.99	1232.01	3	RPKHPINHQGLSPEVLNENLLRFVVAPFPEVF	αs1-casein	NP_001272624.1	16–47(P^31^→L)
3849.31	3852.47	3850.10	642.52	6	RPKHPINHQGLSPEVLNENLLRFVVAPFPEVFR	αs1-casein	NP_001272624.1	16–48(P^31^→L)
3944.68	3943.73	3941.18	1314.4	3	VLPVPQKVVPQRDMPIQAFLLYQEPVLGPVRGPFP	β-casein	P33048	170–204(A177→V) Mox
4054.94	4054.91	4052.28	1013.82	4	LPVPQKVVPQRDMPIQAFLLYQEPVLGPVRGPFPIL	β-casein	P33048	171–206(A^177^→V)
4162.48	4154.05	4151.35	1384.45	3	VLPVPQKVVPQRDMPIQAFLLYQEPVLGPVRGPFPIL	β-casein	P33048	170–206(A^177^→V)
4264.35	4269.18	4266.42	1422.81	3	VLPVPQKVVPQRDMPIQAFLLYQEPVLGPVRGPFPILV	β-casein	P33048	170–207(A^177^→V) M^ox^
4356.77	4353.3	4350.49	1088.37	4	KVLPVPQKAVPQRDMPIQAFLLYQEPVLGPVRGPFPILV	β-casein	P33048	169–207
4810.2	4810.78	4807.70	1202.68	4	SLSQPKVLPVPQKVVPQRDMPIQAFLLYQEPVLGPVRGPFPIL	β-casein	P33048	164–206(A^177^→V) M^ox^;
								163–205(A^177^→V) M^ox^
4922.04	4923.94	4920.79	1230.96	4	LSLSQPKVLPVPQKVVPQRDMPIQAFLLYQEPVLGPVRGPFPIL	β-casein	P33048	163–206(A^177^→V) M^ox^
5017.09	5023.08	5019.86	1004.77	5	VLSLSQPKVLPVPQKVVPQRDMPIQAFLLYQEPVLGPVRGPFPIL	β-casein	P33048	162–206(A^177^→V) M^ox^
5107.34	5109.13	5105.87	1021.98	5	QSVLSLSQPKVLPVPQKVVPQRDMPIQAFLLYQEPVLGPVRGPFPI	β-casein	P33048	160–205(A^177^→V);
5192.21	5181.23	5177.92	1036.37	5	SVLSLSQPKVLPVPQKAVPQRDMPIQAFLLYQEPVLGPVRGPFPILV	β-casein	P33048	161–207 M^ox^
5353.01	5352.39	5348.99	1070.58	5	QSVLSLSQPKVLPVPQKVVPQRDMPIQAFLLYQEPVLGPVRGPFPILN	β-casein	P33048	160–207(A^177^→V, V^207^→N) M^ox^
5828.2	5829.95	5826.10	1166.07	5	LVQSWMHQPPQPLSPTVMFPPQSVLSLSQPKVLPVPQKAVPQRDMPIQAFL	β-casein	P33048	138–189 M^ox^
5914.71	5911.13	5907.28	1182.30	5	TVMFPPQSVLSLSQPKVLPVPQKVVPQRDMPIQAFLLYQEPVLGPVRGPFPIL	β-casein	P33048	154–206(A^177^→V) M^ox^
6001.46	5998.21	5994.31	1199.74	5	TVMFPPQSVLSLSQPKVLPVPQKAVPQRDMPIQAFLLYQEPVLGPVRGPFPILV	β-casein	P33048	154–207 2M^ox^
6279.61	6279.56	6275.48	1255.88	5	LSPTVMFPPQSVLSLSQPKVLPVPQKAVPQRDMPIQAFLLYQEPVLGPVRGPFPILV	β-casein	P33048	151–207 M^ox^

**Table 3:** Bioinformatic analysis for prediction of possible functions of differentially represented milk peptides here identified in mastitic goat samples. Prediction was performed as reported in the experimental section. Reported are prediction score from: (i) CAMPR3 software and (ii) AMP scanner software (for antimicrobial); (iii) dPABBs software (for antibiofilm); (iv) Antiinflam software (for antiinflammatory); (v) AVPPRED software (for composition model—CM and physiochemical model—PM) (for antiviral). Highlighted are prediction scores having numerical values above the threshold limits.

Peptide	CAMP_R3_ Score	AMP Scanner Score	dPABBs Score	AntiInflam Score	AVP_PRED_ Score (CM; PM)	
LGPVRGPFPI	0.44	0.78	−1.23	−0.70	40.27	19.52
GPVRGPFPILV	0.42	0.79	−0.83	0.90	41.47	18.95
TNAIPYVRYL	0.04	0.75	0.39	2.21	18.16	27.06
VLGPVRGPFPIL	0.53	0.87	−0.76	0.70	43.15	27.98
INHQGLSPEVPN	0.07	0.05	−0.44	−0.17	30.22	9.73
EPVLGPVRGPFPIL	0.07	0.91	−0.95	0.42	42.42	30.30
QEPVLGPVRGPFPIL	0.06	0.88	−0.84	0.31	41.60	28.64
QEPVLGPVRGPFPIL	0.06	0.88	−0.84	0.31	41.60	28.64
QEPVLGPVRGPFPILV	0.06	0.92	−0.56	0.22	42.16	34.05
QEPVLGPVRGPFPILV	0.06	0.92	−0.56	0.22	42.16	34.05
LYQEPVLGPVRGPFPI	0.10	0.68	−0.86	0.29	41.41	28.72
QGWGTFLREAGQGAKDM	0.19	0.73	−0.58	−1.02	45.02	32.29
QGWGTFLREAGQGAKDM	0.19	0.73	−0.58	−1.02	45.02	32.29
YQEPVLGPVRGPFPILV	0.09	0.92	−0.58	0.15	42.09	33.71
LSQPKVLPVPQKVVPQR	0.49	0.08	−0.18	−0.81	42.40	47.45
LLYQEPVLGPVRGPFPILV	0.18	0.61	−0.62	0.76	45.61	47.43
QGWGTFLREAGQGAKDMWR	0.24	1.00	−0.35	−1.01	48.07	46.73
QGWGTFLREAGQGAKDMWR	0.24	1.00	−0.35	−1.01	48.07	46.73
FLLYQEPVLGPVRGPFPILV	0.25	0.76	−0.72	0.67	47.05	49.88
AMKPWTQPKTNAIPYVRYL	0.38	0.98	0.35	0.56	34.79	33.59
PIQAFLLYQEPVLGPVRGPFPILV	0.19	0.65	−0.54	0.39	47.90	63.06
MPIQAFLLYQEPVLGPVRGPFPILV	0.04	0.46	−0.59	0.33	49.54	63.82
DMPIQAFLLYQEPVLGPVRGPFPILN	0.05	0.12	−0.95	0.28	45.14	48.97
AVPQRDMPIQAFLLYQEPVLGPVRGPFPI	0.08	0.01	−0.59	0.30	42.37	63.42
VVPQRDMPIQAFLLYQEPVLGPVRGPFPI	0.08	0.01	−0.44	0.30	42.18	63.75
AVPQRDMPIQAFLLYQEPVLGPVRGPFPIL	0.09	0.01	−0.60	0.65	45.52	63.90
VPQRDMPIQAFLLYQEPVLGPVRGPFPILN	0.07	0.03	−0.66	0.65	44.77	63.89
AVPQRDMPIQAFLLYQEPVLGPVRGPFPIL	0.09	0.01	−0.60	0.65	45.52	63.90
RPKHPINHQGLSPEVLNENLLRFVVAPFPEVF	0.05	0.20	0.06	−0.84	47.89	65.47
RPKHPINHQGLSPEVLNENLLRFVVAPFPEVFR	0.08	0.76	0.17	−0.84	47.52	65.33
PVPQKVVPQRDMPIQAFLLYQEPVLGPVRGPFPILN	0.07	0.01	−0.38	0.39	43.43	64.09
LPVPQKVVPQRDMPIQAFLLYQEPVLGPVRGPFPIL	0.07	0.01	−0.42	0.39	44.96	64.07
VLPVPQKVVPQRDMPIQAFLLYQEPVLGPVRGPFPIL	0.08	0.01	−0.28	0.35	44.82	64.07
VLPVPQKVVPQRDMPIQAFLLYQEPVLGPVRGPFPILN	0.08	0.01	−0.25	0.32	44.97	64.07
KVLPVPQKAVPQRDMPIQAFLLYQEPVLGPVRGPFPILV	0.11	0.02	−0.13	0.29	46.59	64.06
SLSQPKVLPVPQKVVPQRDMPIQAFLLYQEPVLGPVRGPFPIL	0.06	0.01	−0.48	0.09	45.52	64.07
LSLSQPKVLPVPQKVVPQRDMPIQAFLLYQEPVLGPVRGPFPIL	0.06	0.01	−0.50	0.07	46.52	64.08
VLSLSQPKVLPVPQKVVPQRDMPIQAFLLYQEPVLGPVRGPFPIL	0.08	0.01	−0.38	0.05	46.27	64.08
QSVLSLSQPKVLPVPQKVVPQRDMPIQAFLLYQEPVLGPVRGPFPI	0.05	0.01	−0.46	−0.21	44.37	64.08
SVLSLSQPKVLPVPQKAVPQRDMPIQAFLLYQEPVLGPVRGPFPILV	0.07	0.01	−0.43	0.01	46.80	64.08
QSVLSLSQPKVLPVPQKVVPQRDMPIQAFLLYQEPVLGPVRGPFPILN	0.05	0.02	−0.47	−0.01	45.88	64.08
LVQSWMHQPPQPLSPTVMFPPQSVLSLSQPKVLPVPQKAVPQRDMPIQAFL	0.00	0.00	−0.78	−0.49	43.42	64.08
TVMFPPQSVLSLSQPKVLPVPQKVVPQRDMPIQAFLLYQEPVLGPVRGPFPIL	0.01	0.01	−0.57	−0.08	44.99	64.08
TVMFPPQSVLSLSQPKVLPVPQKAVPQRDMPIQAFLLYQEPVLGPVRGPFPILV	0.02	0.01	−0.52	−0.10	45.57	64.08
LSPTVMFPPQSVLSLSQPKVLPVPQKAVPQRDMPIQAFLLYQEPVLGPVRGPFPILV	0.01	0.01	−0.66	−0.13	45.45	64.08

## Supplementary Materials

Supplementary materials are available online at https://www.mdpi.com/2079-7737/9/8/193/s1. Table S1: Enzymes and cleavage patterns used by Enzyme-Predictor, Figure S1: Mass spectrum of a control (A) and a clinical with SCC > 1500 × 103 cells (B) milk sample, Figure S2: Helical wheel representation of differentially represented peptides in mastitic milk samples with respect to control.
