# Supplementary material for: Novel Biomarkers of Mastitis in Goat Milk Revealed by MALDI-TOF-MS-Based Peptide Profiling
Source: Biology (Basel). 2020 Jul 28;9(8):193. doi: 10.3390/biology9080193 (PMC7464427; doi:10.3390/biology9080193)
Supplement: Supplementary file 1 [file biology-09-00193-s001.pdf]

Novel biomarkers of mastitis in goat milk revealed by MALDI-TOF-MS-based peptide profiling

Table S1. Enzymes and cleavage patterns used by Enzyme-Predictor (http://bioware.ucd.ie/~enzpred/Enzpred.php).

Enzyme name	P4	P3	P2	P1	P1'	P2'
Arg-C proteinase	-	-	-	R	-	-
Asp-N endopeptidase	-	-	-	-	D	-
Chymotrypsin-high specificity (C-term to [FYW], not before P)	-	-	-	F or Y	not P	-
	-	-	-	W	not M or P	-
Chymotrypsin-low specificity (C-term to [FYWML], not before P)	-	-	-	F,L or Y	not P	-
	-	-	-	W	not M or P	-
	-	-	-	M	not P or Y	-
	-	-	-	H	not D,M,P or W	-
LysC	-	-	-	K	-	-
Pepsin (pH>2)	-	not H,K or R	not P	not R	F or L	not P
	-	not H,K or R	not P	F or L	-	not P
Proline-endopeptidase	-	-	H,K or R	P	not P	-
Trypsin	-	-	-	K or R	not P	-
	-	-	W	K	P	-
	-	-	M	R	P	-
Modified-chymotrypsin	-	-	-	F,W,Y or L	not P	-
Elastase	-	-	-	A,V,I,L,G or R	G,P,A,L or F	-
Proline-endopeptidase_diff	-	-	-	P	-	-
Plasmin	-	-	-	K or R		-
CathepsinD	-	-	-	A,V,L,I,P,M,F or W	A,V,L,I,P,M or F	-

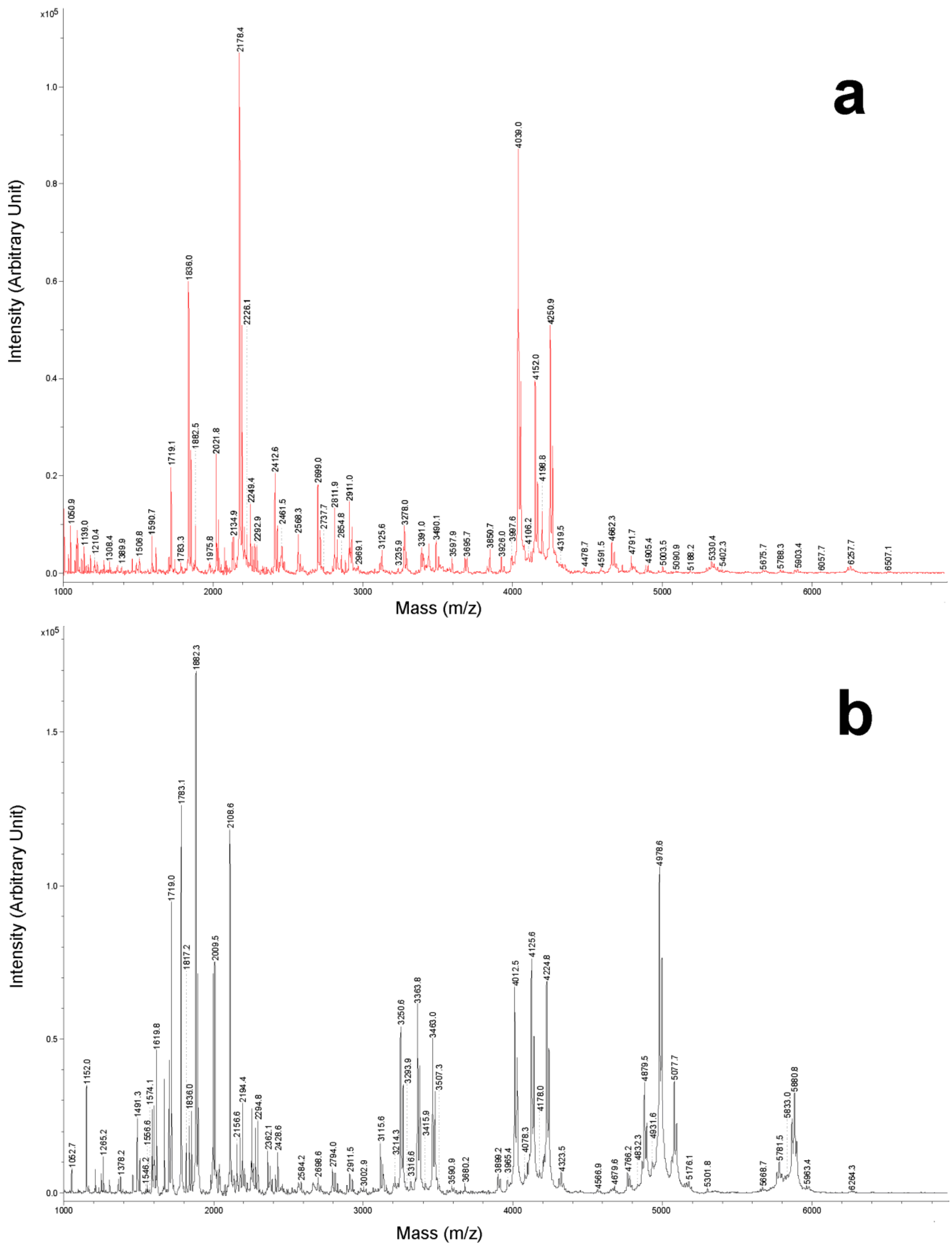

Figure S1. MALDI-TOF mass spectrum acquired for a control milk sample (**a**) and a clinical mastitic (**b**) milk sample with a SCC value > 1500 x 103 cells/ml.

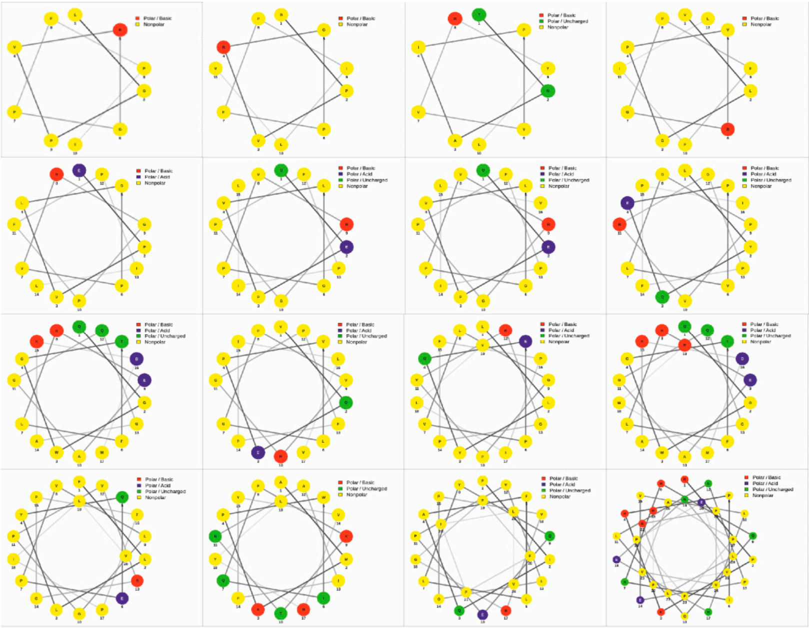

Figure S2. Helical wheel representation of peptides reported in Table 3 as antimicrobial molecules, highlighting their amphipathic character.
